# Dose–response relationship between physical activity and subjective sleep quality in patients with insomnia: a systematic review and Bayesian meta-analysis of randomized controlled trials

**DOI:** 10.1186/s12966-026-01905-0

**Published:** 2026-04-11

**Authors:** Zan Huang, Youzheng Zhang, Wenxin Xu

**Affiliations:** School of Physical Education and Sport Science, Fujian Normal University, Fuzhou, 350117 China

**Keywords:** Insomnia, Subjective sleep quality, Physical activity, Dose–response, Meta-analysis

## Abstract

**Background:**

Insomnia is a prevalent public health concern with significant consequences for well-being. While physical activity (PA) is recognized as a beneficial non-pharmacological intervention, its precise dose-response relationship with subjective sleep quality remains insufficiently quantified. This study aimed to systematically evaluate the efficacy of PA on subjective sleep quality in patients with insomnia and to quantitatively delineate the dose–response relationship using Bayesian meta-analysis.

**Methods:**

A systematic search was conducted across Web of Science, PubMed, Embase, PsycINFO, and the Cochrane Library from inception to February 2026. Randomized controlled trials (RCTs) investigating the effects of PA on subjective sleep quality in participants diagnosed with insomnia were included. A Bayesian random-effects meta-analysis was performed to calculate pooled effect sizes (Hedges’g) with 95% Credible Intervals (CrI). Dose–response relationships were modelled using restricted cubic splines to identify minimum and optimal dosages. Dosage was expressed as the weekly volume in MET-minutes (Metabolic Equivalent of Task × minutes/week). The certainty of evidence was assessed using the GRADE approach.

**Results:**

24 RCTs involving 1,591 participants were included. Bayesian synthesis indicated low evidence that PA significantly improves subjective sleep quality compared with inactive controls (Hedges’g = -0.56, 95% CrI: -0.68, -0.45). Dose–response analysis revealed a non-linear “L-shaped” relationship. Benefits accrued rapidly at lower volumes, with a minimum effective dose estimated at 100 METs-min/week. The therapeutic effect peaked at an optimal dose of approximately 650 METs-min/week (Hedges’g = -0.64, 95% CrI: -0.85, -0.44), beyond which additional volume yielded diminishing marginal returns. Moderation analyses indicated that the effect was more pronounced in adults (Hedges’ g = -1.00) than in older adults (Hedges’ g = -0.51), and was consistent across different diagnostic methods and measurement instruments. Various types of exercise, including aerobic, strength, mind-body, and multicomponent training, all demonstrated beneficial effects.

**Conclusions:**

Physical activity is an effective treatment for insomnia, characterized by an L-shaped dose–response curve. The relationship is characterized by a rapid initial benefit at low volumes, with a statistically significant response observed at 100 MET-min/week and a plateau in effect occurring at approximately 650 MET-min/week, beyond which additional volume yields diminishing returns.

**Trial registration:**

CRD420251172873.

**Supplementary Information:**

The online version contains supplementary material available at 10.1186/s12966-026-01905-0.

## Background

Sleep disorders, particularly insomnia, have emerged as a critical global public health concern, affecting up to 30% of the adult population and posing a significant threat to individual quality of life, daytime functioning, and both physical and mental well-being [1]. According to recent assessments of the global prevalence and burden of insomnia, approximately 16% of adults worldwide meet the diagnostic criteria for insomnia [2]. Insomnia is not only inextricably linked to mental health conditions such as depression and anxiety [3] but has also been established as an independent risk factor for a range of chronic diseases, including cardiovascular disease, type 2 diabetes, obesity, and cognitive decline [4].

Currently, the first-line evidence-based treatment for chronic insomnia is Cognitive Behavioral Therapy for Insomnia (CBT-I) [5, 6]. By targeting the underlying cognitive and behavioral factors contributing to insomnia, CBT-I yields durable therapeutic effects, and its efficacy is well-documented [7]. However, a substantial gap exists in the implementation of CBT-I in clinical practice. Limitations—including a severe shortage of trained professionals, high out-of-pocket costs for patients, and the significant time commitment required for the standard 4–8 sessions—have resulted in extremely low utilization rates [8–10]. Given the poor accessibility of CBT-I, pharmacotherapy, such as benzodiazepines or Z-drugs, remains the default option for the vast majority of patients [5]. While these pharmacological agents provide short-term symptom relief, their long-term use in chronic insomnia management is restricted by issues of dependence, tolerance, cognitive impairment, and other adverse effects [11, 12].

Among non-pharmacological interventions, physical activity (PA) defined as any bodily movement produced by skeletal muscles that requires energy expenditure [13], represents a highly effective and promising yet not fully characterized strategy, garnering attention for its cost-effectiveness, high accessibility, and minimal side effects [14, 15]. A substantial and growing body of evidence confirms that regular physical activity is an effective intervention for improving sleep in patients with insomnia [16–18]. A network meta-analysis indicated that exercise provides sustained, long-term improvements in sleep quality comparable to those of CBT-I when compared to control conditions [19]. Furthermore, a recent randomized non-inferiority trial demonstrated that tai chi, a mind-body exercise, was non-inferior to CBT-I for the long-term management of chronic insomnia in middle-aged and older adults [20]. Consequently, several clinical guidelines now recommend exercise interventions as an adjunctive therapy for treating insomnia symptoms [12, 21].

However, while the efficacy of physical activity is well-established, the dose–response relationship between PA and subjective sleep quality in patients with insomnia has been explored only to a limited extent [22, 23]. Although a recent large-scale network meta-analysis by Wang et al. provided a reference of 920 METs-min/week for the general population, and Xue et al. suggested a low-intensity recommendation of approximately 440 METs-min/week for older adults, the precise dose–response relationship for the core clinical population—diagnosed insomnia patients—remains insufficiently elucidated. Patients with insomnia often exhibit hyperarousal of the hypothalamic-pituitary-adrenal axis and autonomic dysregulation. Consequently, their physiological adaptation curve to exercise load may differ fundamentally from that of healthy individuals. This implies that the threshold, optimal window, and upper limit of exercise stimulation for this population may be distinct from those of healthy adults or the general elderly population. For insomnia patients specifically, determining the minimum and optimal dosage to improve subjective sleep quality remains a central, unresolved question.

Therefore, the primary objective of this study is to bridge this critical knowledge gap by quantitatively delineating the dose–response relationship between physical activity and subjective sleep quality improvements in patients with insomnia. Clarifying this dose–response curve is a crucial step in translating physical activity research into clinical practice guidelines for insomnia. The findings will provide a robust, scientific basis for evaluating exercise interventions, offering valuable, evidence-based guidance for clinicians and patients alike.

## Methods

### Registration

The protocol for this systematic review was registered with the International Prospective Register of Systematic Reviews (PROSPERO) (Registration number: CRD420251172873) to ensure transparency and minimize reporting bias. This manuscript is reported in accordance with the Preferred Reporting Items for Systematic Reviews and Meta-Analyses (PRISMA) 2020 statement [24]. The complete PRISMA 2020 checklist is provided in the Supplementary Materials.

### Information sources and search strategy

A systematic literature search was conducted across Web of Science, PubMed, Embase, Scopus, PsycINFO, and the Cochrane Library. The search spanned from the inception of each database to 23 August 2025, with an updated search conducted on 1 February 2026. To ensure comprehensive coverage, the reference lists of included studies and relevant systematic reviews were manually screened. The search strategy combined Medical Subject Headings (MeSH) and free-text terms using Boolean operators to identify studies concerning physical activity and subjective sleep quality. Specific search strings were tailored to the syntax rules of each database. No language restrictions were imposed during the search phase. Detailed search strategies for each database are presented in the Supplementary Materials.

### Eligibility criteria

Study selection was based on the “Population, Intervention, Comparator, Outcome, and Study Design” (PICOS) framework: (1) Participants: Patients with insomnia, defined as adults (aged ≥ 18 years) with a clinical diagnosis of insomnia according to established criteria (e.g., the Diagnostic and Statistical Manual of Mental Disorders, DSM-5, or the International Classification of Sleep Disorders, ICSD) [25–27] or those exceeding clinical cut-off scores on standardized insomnia scales (e.g., Insomnia Severity Index > 14 [28] or Pittsburgh Sleep Quality Index > 5 [29]); (2) Interventions: Any form of structured physical activity or exercise program (e.g., aerobic exercise, resistance training, mind-body exercise). Studies were required to have a minimum intervention duration of 4 weeks and to provide sufficient detail to quantify the intervention dose, including frequency (sessions/week), intensity (e.g., percentage of maximum heart rate, rating of perceived exertion, metabolic equivalents), and duration (minutes/session) to allow for the calculation of total exercise volume; (3) Comparators: The comparators should include inactive controls (e.g., no intervention, waitlist), attention controls (e.g., sleep education), and light exercise (e.g., stretching). The latter was analyzed as a intervention arm rather than an inactive control; (4) Outcomes: The primary outcome was the change in subjective sleep quality from baseline to post-intervention, measured via validated continuous scales. This review focused exclusively on subjective measures, as they capture the patient’s perception of sleep, which is the core diagnostic criterion for insomnia disorder and is directly relevant to the complaint of poor sleep. The Pittsburgh Sleep Quality Index (PSQI) was the preferred instrument, though other validated scales assessing subjective sleep disturbance, such as Insomnia Severity Index (ISI), were included. Substantial evidence indicates that these instruments exhibit high convergent validity and are significantly correlated in populations with insomnia [30, 31]. Studies were required to report quantitative data (Mean±SD) for both baseline and post-intervention time points; (5) Study Design: To ensure the highest level of evidence, only parallel-group or cluster randomized controlled trials (RCTs) were considered eligible.

Studies were excluded if they: (1) involved participants with significant psychiatric comorbidities (e.g., major depressive disorder, bipolar disorder, or schizophrenia) or secondary insomnia attributable to other medical conditions, substance use, or medications; (2) utilized multi-component interventions (e.g., exercise combined with cognitive therapy) where the isolated effect of physical activity could not be determined; (3) did not report sufficient data (e.g., means, standard deviations, sample sizes) for effect size calculation or dose extraction; (4) were not RCTs (e.g., protocols, conference abstracts, or other non-randomized study designs).

### Study selection and data extraction

All retrieved records were imported into EndNote. Two reviewers independently screened titles and abstracts to assess initial eligibility. Subsequently, full-text articles of potentially relevant studies were retrieved and assessed independently. Discrepancies at any stage were resolved through discussion or, if necessary, adjudication by a third senior researcher. Data were extracted using a pre-piloted, standardized extraction form. Two reviewers independently extracted the following data from each included study: study characteristics (first author, publication year, country, design, sample size), participant characteristics (age, sex, diagnostic criteria, baseline insomnia severity), intervention details (type, frequency, intensity, duration, and total intervention period), and outcome data (mean difference, standard deviation, and sample size). Where data were not directly reported, values were calculated from change scores or other available statistics.

### Data coding and management

Key variables were systematically coded to ensure consistency in effect size calculation and subsequent moderation analyses. Specific modalities were categorized as aerobic exercise, resistance training, yoga, traditional Chinese mind-body exercises, or multi-component interventions (primarily combining aerobic and resistance training). regarding participant characteristics, age (adults vs. older adults, adults were defined as those with a mean age < 60 years; older adults were defined as those with a mean age ≥ 60 years), baseline sleep disturbance severity (mild vs. moderate, based on the primary scale used in the study, mild was defined as an ISI score of 8–14 or a PSQI score of 6–8; moderate-to-severe was defined as an ISI score ≥ 15 or a PSQI score ≥ 9), diagnostic method (Studies were categorized based on whether participants were recruited via strict diagnostic criteria (DSM/ICSD) or via questionnaire cut-off scores), and sex distribution were coded for moderation analysis.

To calculate the dose–response relationship for specific exercise modalities, the total weekly volume of physical activity was quantified as the product of its Metabolic Equivalent of Task (MET) value, session duration, and weekly frequency, expressed in MET-minutes/week—a standardized metric for estimating total energy expenditure from physical activity. This approach aligns with established methodologies in exercise physiology and epidemiology for dose quantification [32–34]. METs were calculated using reported intensity metrics (e.g., heart rate, VO_2max_, HR_max_). Where these were unavailable, estimates were made based on the reported activity type using the *Adult Compendium of Physical Activities* [35, 36]. All variables were coded independently by two researchers, with cross-checking employed to resolve discrepancies and ensure data accuracy.

### Risk of bias assessment

Two reviewers independently assessed the risk of bias for each included RCT using the Cochrane Risk of Bias tool version 2 (RoB 2) [37]. The RoB 2 tool evaluates bias across five domains: (1) bias arising from the randomization process; (2) bias due to deviations from intended interventions; (3) bias due to missing outcome data; (4) bias in measurement of the outcome; and (5) bias in selection of the reported result. For each domain, risk was judged as “Low risk,” “Some concerns,” or “High risk” based on responses to signaling questions. An overall risk of bias judgement was determined for the primary outcome of each study. Disagreements were resolved through consensus.

### Data synthesis

#### Effect size calculation

We adopted an arm-based approach, wherein each study arm (e.g., a specific physical activity intervention or the control condition) constitutes a separate row in the dataset. For multi-arm trials, this structure naturally accommodates all arms, with each arm’s sample size, mean outcome, and standard deviation recorded independently. The Standardized Mean Difference (SMD) was used as the effect size metric for continuous variables. Hedges’ g correction was applied to mitigate small-sample bias. Calculations were performed in R version 4.5.0 (R Core Team, 2013) using the *escalc()* function within the *metafor* package [38].

#### Bayesian meta-analysis

A Bayesian approach was employed for data synthesis. This method integrates prior information (priors) with Markov Chain Monte Carlo (MCMC) simulations to compute the probability distribution of effect sizes, effectively handling heterogeneity and uncertainty between studies. Bayesian random-effects models were fitted using the brms package in R [39]. The effect size for each study was represented by the post-intervention Hedges’g between the intervention and control groups. We utilized weakly informative priors: a normal distribution *(0*,* 1)* for the overall intercept and a Half-Cauchy distribution *(0*,* 1)* for the between-study heterogeneity parameter *tau* [40]. Each model was estimated using four Markov chains, with 10,000 iterations per chain. Model convergence was assessed by verifying that the potential scale reduction factor (*Rhat*) for all parameters was < 1.01, combined with visual inspection of trace plots. Heterogeneity was quantified by examining the posterior distribution of the random-effects standard deviation (parameter *tau*).

#### Dose–response analysis

To investigate the precise relationship between physical activity dose and improvements in subjective sleep quality, we modelled the relationship between weekly physical activity volume and subjective sleep quality using both linear and non-linear terms (natural splines, restricted cubic splines, and polynomials). Model selection was based on the Leave-One-Out Information Criterion (LOOIC). The predicted response was reported as Hedges’g with 95% Credible Intervals (CrI) to assess the certainty of estimates. We estimated the optimal physical activity dose for improving subjective sleep quality in insomnia patients—defined as the dose at which the predicted beneficial effect was maximized. Results indicated that a restricted cubic spline model with 4 knots provided the best fit for the data.

#### Publication bias and sensitivity analysis

Publication bias was evaluated by visually inspecting the symmetry of funnel plots and conducting Egger’s regression test. A symmetric inverted funnel distribution suggests no serious publication bias, whereas asymmetry indicates potential bias. An Egger’s test two-tailed p-value > 0.05 indicated the absence of severe publication bias [41]. If significant bias was detected, the trim-and-fill method was applied [42]. To assess robustness, a sensitivity analysis was conducted by excluding studies with a “High risk” of bias to evaluate the stability of both the pooled effect estimate and the dose–response curve shape.

#### Certainty of evidence

The overall certainty of evidence for primary outcomes was assessed using the Grading of Recommendations Assessment, Development and Evaluation (GRADE) approach. Two reviewers performed the evaluation independently. As all included studies were RCTs, the initial certainty was rated as “High.” Evidence could be downgraded based on five domains: (1) risk of bias; (2) inconsistency (heterogeneity); (3) indirectness; (4) imprecision; and (5) publication bias. Each domain could lead to downgrading by one level (serious concern) or two levels (very serious concern). The final certainty of evidence was rated as High, Moderate, Low, or Very low. All judgements were documented with supporting rationales.

## Results

### Study selection

**Fig. 1:**
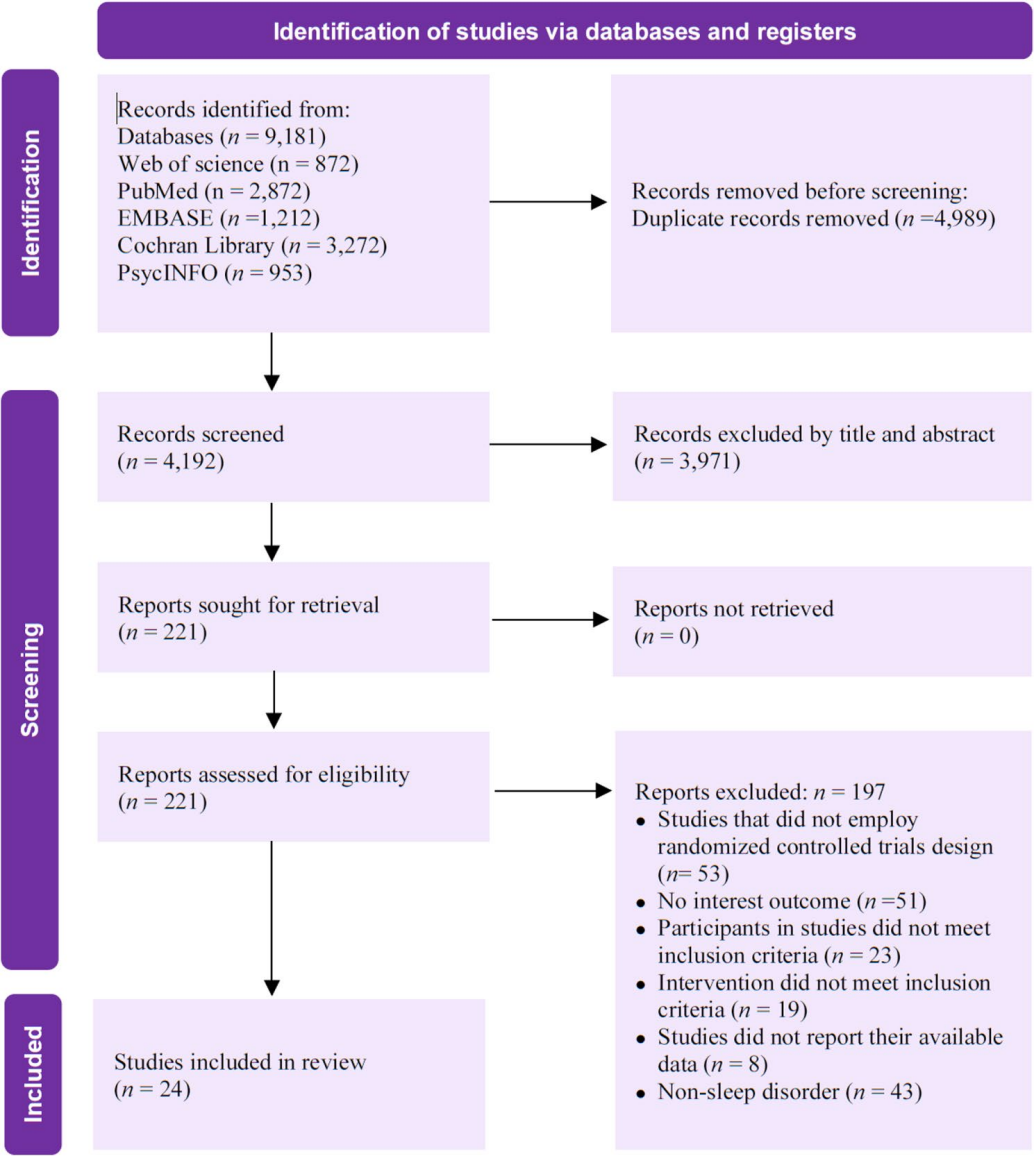
PRISMA flow diagram

The initial literature search was conducted in strict accordance with the pre-defined strategy. A systematic search across five electronic databases, including Web of Science, PubMed, EMBASE, PsycINFO, and the Cochrane Library, yielded a total of 9,181 records. After importing all records into EndNote citation management software, the system automatically identified and removed 4,989 duplicates, leaving 4,192 unique records for the initial screening phase. Two researchers independently screened the titles and abstracts of these records. At this stage, 3,971 records were excluded as they were clearly irrelevant to the study topic. Following the initial screening, 221 potentially eligible articles were identified, and their full texts were retrieved for a detailed eligibility assessment. During the full-text review, two researchers again independently and rigorously assessed these articles. Ultimately, 197 studies were excluded for failing to fully meet the inclusion criteria. A total of 24 studies fully satisfied all inclusion criteria and were included in the final analysis. The detailed process of study selection is clearly presented in the PRISMA 2020 flow diagram (Fig. 1).

### Characteristics of included studies

The baseline characteristics of the 24 randomized controlled trials (RCTs) included in this review are summarized in Table 1. The publication dates of these studies ranged from 2004 to 2025. The studies involved a total of 1,591 participants diagnosed with insomnia, with 843 allocated to intervention groups and 748 to control groups. Geographically, the studies were predominantly conducted in North America (*n* = 4) and Asia (*n* = 12), followed by Europe (*n* = 3), South America (*n* = 4), and Africa (*n* = 1). Sample sizes varied considerably across studies, ranging from 17 to 250 participants. Regarding participant demographics, the reported mean age ranged from 43.7 to 75.4 years. All studies reported a high proportion of female participants, ranging from 55% to 100%. Diagnostic criteria for insomnia varied: 8 studies utilized criteria from the DSM (including editions IV and 5); 2 studies used the ICSD-3; and the remaining studies screened participants based on clinical cut-off scores from validated questionnaires. Intervention modalities were diverse and categorized as follows: aerobic exercise (*n* = 9), such as brisk walking or cycling; traditional Chinese mind-body exercises (*n* = 6), such as Tai Chi, Qigong and Baduanjin; yoga (*n* = 2); resistance training (*n* = 3); and multi-component exercise combining aerobic and resistance training (*n* = 4). The total duration of interventions ranged from 4 to 24 weeks. The control conditions across the included studies comprised waitlist control (*n* = 7), treatment as usual (*n* = 8), health education (*n* = 7), and light exercise (*n* = 2). For analysis, the light exercise groups were treated as intervention arms. Regarding outcome measures, subjective sleep quality was the core outcome in all studies.

**Table 1:** Characteristics of included studies

Study (Author, Year)	Country	Sample Size (Int/Ctrl)	Mean Age (years)	Female (%)	Diagnostic Criteria	Intervention Details	Control Condition	Outcome Measures
Dadgostar 2023[43]	Iran	16/16	43.7	NA	ICSD-3	Participants completed a combined aerobic and resistance training program six times per week for 45 min per session over 12 weeks. Resistance training targeted major muscle groups at a progressive intensity from 50% to 70% of 10-RM, while aerobic exercise consisted of moderate-intensity walking (60% heart rate reserve). Sessions were supervised, and adherence was logged.	Waitlist control	PSQI
Baron 2023[44]	France	12/12	46.4	100	DSM-5	The intervention involved supervised aerobic walking sessions three times per week for 75 min each. Each session combined 50 min of moderate-intensity (70–80% of maximum heart rate) and 25 min of vigorous-intensity (> 80% of maximum heart rate) exercise, monitored individually with heart rate sensors.	Treatment as usual	ISI
Siu 2021[45]	China	105/110	66.5	80	DSM-5	This study compared two interventions: a Tai Chi group practicing the Yang-style 24-form and a conventional exercise group performing brisk walking and muscle-strengthening. Both groups attended three supervised one-hour sessions per week in small groups for 12 weeks.	Waitlist control	PSQI, ISI
Khalsa 2021[46]	USA	20/20	43.5	55	PSQI > 5	Participants were assigned a daily 45-minute home-based Kundalini yoga practice for 8 weeks, focusing on seated meditation, breathing techniques, and relaxation. They received an initial in-person training session and were instructed to practice in the evening before bedtime.	Health education	PSQI, ISI
Fan 2020[47]	China	67/72	70.3	82.1	PSQI > 5	The intervention consisted of a group-based Baduanjin Qigong program, practiced five times per week for 45 min per session over 24 weeks. Sessions were guided by trained tutors, and attendance was recorded.	Waitlist control	PSQI
Brandao 2018[48]	Brazil	61/64	69.9	88.1	PSQI > 5	Participants followed a multicomponent home exercise program (including aerobic, strength, balance, and flexibility exercises) three times per week for 40 min. The program was unsupervised but guided by biweekly home visits, with intensity self-adjusted to a Borg scale rating of 13–15.	Health education	PSQI
Yeung 2018[49]	China	18/19	49.8	88.9	DSM-5	The “Zero-time Exercise” intervention trained participants to integrate physical activity into daily life. After two initial 2-hour group training sessions, participants were to practice these brief exercises daily.	Health education	ISI
D’urea 2019[50]	Brazil	10/8	44.5	NA	DSM-5	A supervised, lab-based resistance training program was conducted three times per week for 50 min per session over 4 months. The intensity was progressively increased from 50% to 60% of 1-RM, targeting all major muscle groups.	Treatment as usual	ISI, PSQI
Afonso 2011[51]	Brazil	15/15	NA	NA	DSM-4	The yoga intervention comprised two supervised one-hour group sessions per week for 16 weeks. The practice included specific asanas and bellows breathing (bhastrika) techniques, ending with directed relaxation.	Waitlist control	ISI
Cai 2014[52]	China	10/9	59.3		PSQI > 5	Participants engaged in supervised, group-based step aerobics three times per week for 40–45 min at a moderate-to-vigorous intensity (75–85% of heart rate reserve) for 10 weeks.	Treatment as usual	PSQI
Reid 2010[53]	USA	10/7	62	0	PSQI > 5	The intervention was a supervised aerobic exercise program (walking, cycling, or treadmill) conducted four times per week in the afternoon or evening. After a 4–6 week conditioning phase, participants exercised at 75% of maximum heart rate for 30–40 min per session for the remainder of the 16-week study.	Waitlist control	PSQI
King 2008[54]	USA	32/27	61.9	66.6	PSQI > 5	Participants were asked to complete five exercise sessions per week: two supervised group classes and three home-based sessions, each lasting 30–40 min, over 12 months. The primary activity was moderate-to-vigorous intensity aerobic exercise (60–85% of peak heart rate), monitored with heart rate monitors.	Health education	PSQI
Yeung 2025[55]	China	70/70	48	81.4	DSM-5	This exercise program involved two initial 2-hour training sessions a week apart, teaching participants how to integrate short bouts of exercise into daily routines. Practice was supported by twice-weekly phone calls during the first 8 weeks of the 24-week study period.	Health education	ISI
Cunha 2025	Brazil	35/34	68.2	NA	PSQI > 5	A supervised, facility-based resistance training program was performed three times per week for 60 min. Participants performed 3 sets of 10–15 repetitions for eight whole-body exercises, with training loads progressively adjusted based on performance.	Treatment as usual	PSQI
Chen 2024[56]	China	37/30	61.6	89.2	DSM-5	The intervention was a supervised, group-based program that combined Tai Chi elements (Chan Si Gong, Zhuang Gong, Chen-style 8-form). Sessions were conducted three times per week for one hour over 12 weeks.	Waitlist control	ISI, PSQI
Irwin 2008[57]	USA	30/22	69.7	73.3	PSQI > 5	Participants received group instruction in Tai Chi Chih three times per week for 40 min per session over 16 weeks. Certified instructors taught the 20-movement set in a graduated manner.	Health education	PSQI
Tseng 2020[58]	China	20/20	61.1	75	PSQI > 5	The program consisted of supervised, graded treadmill walking sessions three times per week for 50 min. Each session included warm-up, 30 min of training at 50–60% of VO2peak, and cool-down, conducted in a laboratory setting.	Waitlist control	PSQI
Massoud 2023[59]	Egypt	28/28	57.4	100	PSQI > 5	A supervised resistance training program using bodyweight and closed-chain exercises was performed three times per week for 6 weeks. The duration, number of sets, and hold time for exercises increased progressively from week to week.	Treatment as usual	PSQI
Gupta 2023[60]	India	18/18	67.5	66.5	PSQI > 5	The combined training included four supervised sessions per week, each lasting 45 min over 4 weeks. Each session comprised 15 min of aerobic walking at moderate intensity (RPE 5–6) and 15 min of resistance exercises at 50% of 1-RM.	Treatment as usual	PSQI
Milot 2025[61]	France	8/9	70.2	76.9	ICSD-3	A multicomponent exercise program (aerobic and resistance) was delivered via live videoconferencing to participants’ homes, supervised by a physical trainer. Sessions lasted one hour, 2–3 times per week for 12–16 weeks, with intensity progressively increased from 60% to 75% of heart rate reserve.	Health education	PSQI, ISI
Chen 2012[62]	China	20/15	71.7	65.3	PSQI > 5	Participants were instructed to perform a 30-minute Baduanjin Qigong routine at home three times per week for 12 weeks. They received initial in-person training, a demonstration video, a brochure, and adherence support through twice-weekly phone follow-ups and logs.	Treatment as usual	PSQI
Chin 2022[63]	China	9/9	62.7	94.4	DSM-5	This study tested different frequencies and intensities of supervised treadmill walking. Participants were assigned to either moderate-intensity (3.25 METs) or vigorous-intensity (6.5 METs) walking, completed in either one longer or three shorter sessions per week, all meeting WHO activity guidelines over 12 weeks.	stretching exercises	PSQI, ISI
Hartescu 2015[64]	UK	17/18	59.8	73.2	PSQI > 5	Participants were instructed to engage in self-directed, moderate-intensity brisk walking for at least 30 min per day on at least five days per week. This followed a 4-week conditioning period, totaling at least 150 min of activity per week for the 4-week intervention.	Treatment as usual	ISI
Li 2004[65]	China	32/42	75.4	48	PSQI > 5	This study compared a Tai Chi group practicing a simplified Yang-style form to a low-impact exercise control group. Both groups attended three supervised, one-hour group sessions per week for 24 weeks, with sessions incorporating music and social interaction.	Low-impact exercise	PSQI

DSM-IV/5: Diagnostic and Statistical Manual of Mental Disorders, 4th/5th Edition; ICSD-3: International Classification of Sleep Disorders, 3rd Edition

*Int* Intervention group, *Ctrl* Control group, *ISI* Insomnia Severity Index, *NA* Not available, *PSQI* Pittsburgh Sleep Quality Index

### Risk of bias assessment

**Fig. 2:**
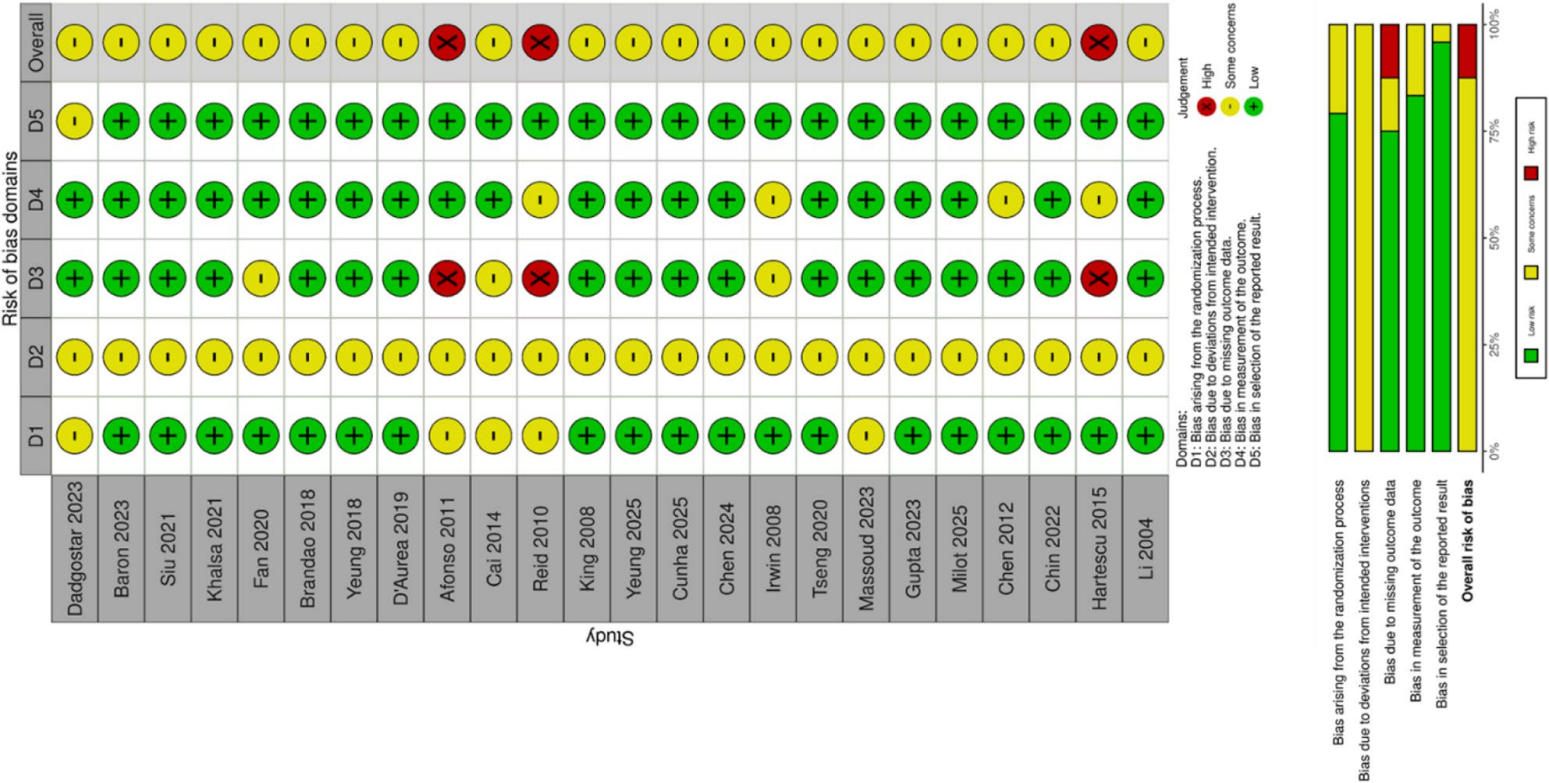
Risk of bias results

The methodological quality of the 24 included RCTs was systematically assessed using the Cochrane Risk of Bias tool version 2 (RoB 2), with assessments completed independently by two researchers who reached consensus. Overall, 21 studies (87.5%) were judged as having some concerns and 3 (12.5%) were rated as high risk. The specific assessment results and summary of risk of bias distribution across domains for all studies are presented in Fig. 2. Analysis of specific domains indicated that bias in selection of the reported result (Domain 5) and bias in measurement of the outcome (Domain 4) were the areas with the lowest risk, with all or most studies performing well. Risk of bias arising from the randomization process (Domain 1) was also generally low, although a small number of studies raised some concerns in these areas. Bias due to deviations from intended interventions (Domain 2) was assessed as raising some concerns across all studies, an expected finding in trials of physical activity interventions where blinding of participants is typically not feasible. The most notable source of bias was bias due to missing outcome data (Domain 3). This was the only domain to yield High risk ratings, which directly resulted in the High overall risk of bias judgements for the studies by Afonso [51], Reid [53] and Hartescu [64].

### Data synthesis and meta-analysis results

#### Overall effect

**Fig. 3:**
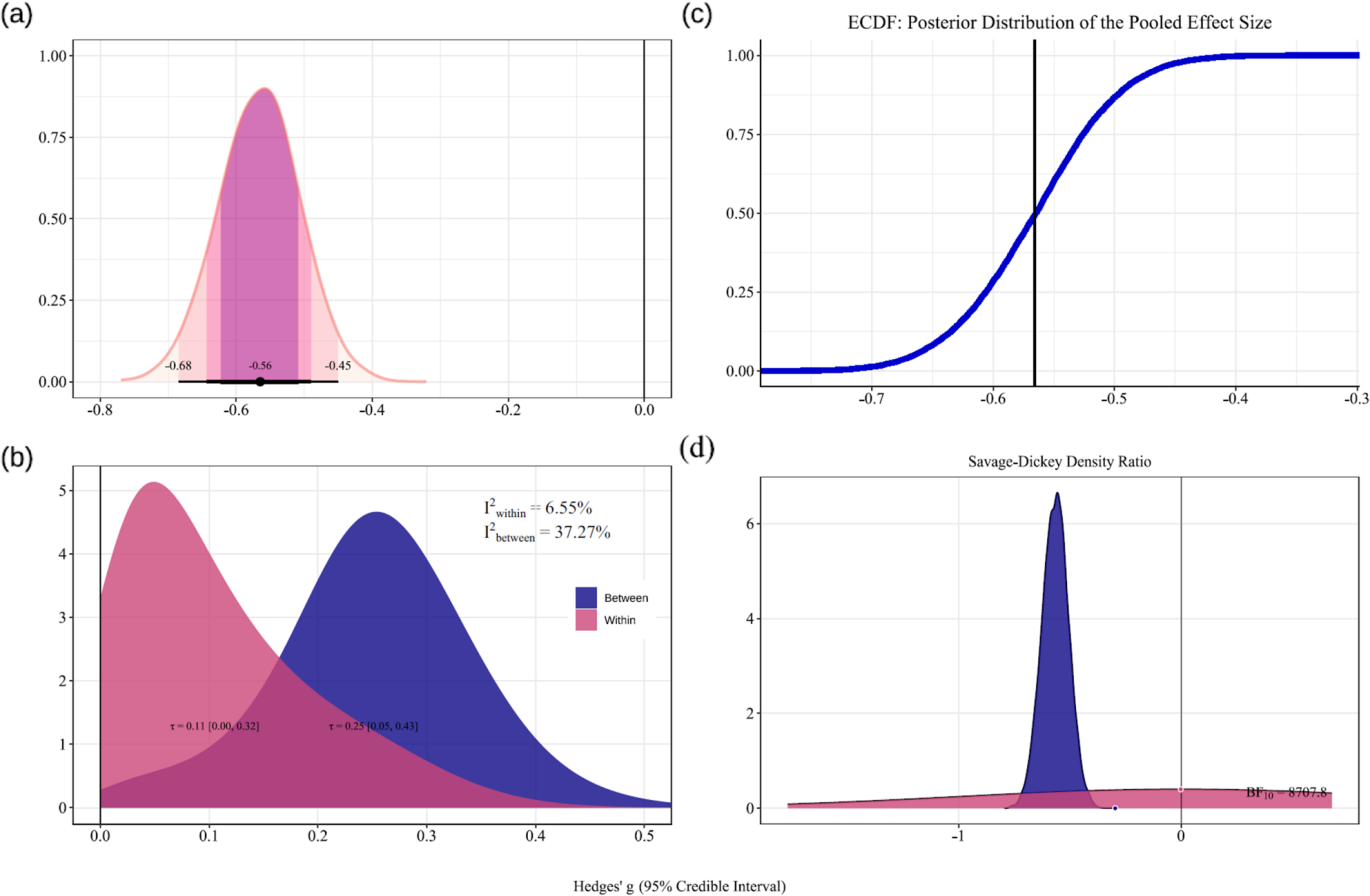
The overall effect of physical activity on subjective sleep quality. (**a**) Main effect. (**b**) Heterogeneity. (**c**) Posterior distribution probability. (**d**) Savage-dickey ratio

A Bayesian random-effects meta-analysis was conducted on data from all 24 studies. The results indicate that, irrespective of dosage differences, there is moderate evidence that physical activity interventions significantly improve subjective sleep quality in patients with insomnia compared to inactive controls (Fig. 3). The overall pooled effect size was Hedges’g = -0.56 (95% CrI: -0.68, -0.45). The posterior probability distribution indicates a 99.9% probability that the effect size is less than 0, with a 87.5% probability that the effect falls between − 0.5 and 0. The analysis also revealed significant heterogeneity between studies. Within-study heterogeneity was low (τ_*within*_ = 0.11; 95% CrI = [0.00, 0.32]; I^2^_*within*_ = 6.55%), and between-study heterogeneity was also low (τ_between_ = 0.25; 95% CrI = [0.05, 0.43]; I^2^_*between*_ = 37.27%).

#### Moderation analysis

**Fig. 4:**
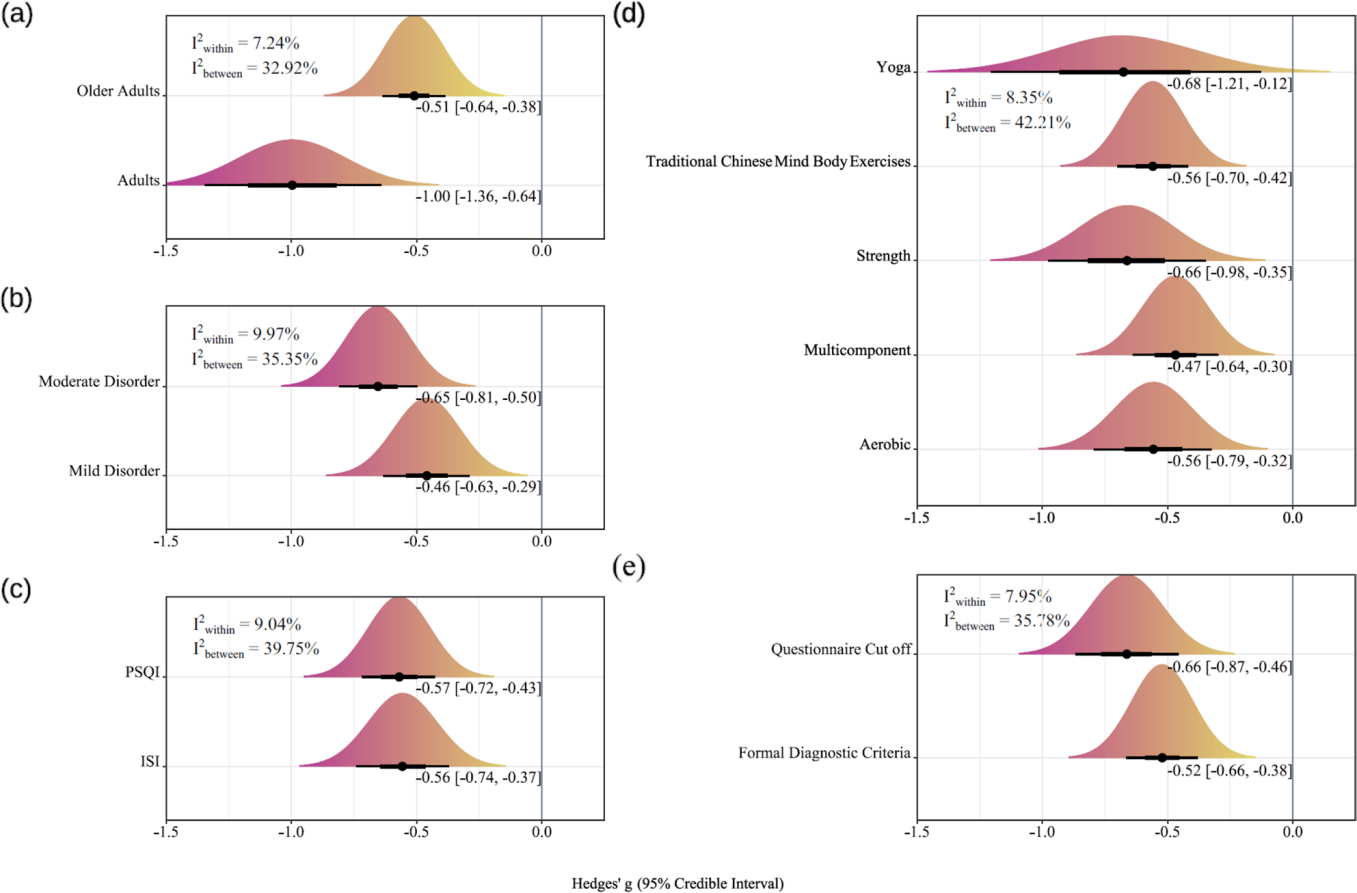
Moderation analysis. (**a**) Age. (**b**) Baseline insomnia severity. (**c**) Measurement instrument. (**d**) Type of physical activity. (**e**) Diagnostic method

The results of moderation analysis are presented in Fig. 4. Subgroup analysis by age demonstrated that the effect of physical activity was Hedges’g = -1.00 (95% CrI: -1.36, -0.64) for adults, indicating a moderate-to-large positive effect. In contrast, the effect size for older adults was − 0.51 (95% CrI: -0.64, -0.38). Analysis based on the method of diagnostic criteria showed an effect size of Hedges’g = -0.52 (95% CrI: -0.68, -0.38) for studies using formal diagnostic criteria (DSM/ICSD), and Hedges’g = -0.66 (95% CrI: -0.87, -0.46) for those defining cases by questionnaire cut-off. Regarding baseline insomnia severity, physical activity yielded an effect size of Hedges’g = -0.46 (95% CrI: -0.63, -0.29) for patients with mild insomnia, and Hedges’g = -0.65 (95% CrI: -0.81, -0.50) for those with moderate insomnia. Furthermore, the percentage of female participants did not significantly moderate the relationship between physical activity and subjective sleep quality (*β* = 0.22, 95% CI [-0.05, 0.50]). Regarding the effectiveness of different physical activity modalities, all types demonstrated beneficial effects on subjective sleep quality compared to control conditions. Strength training showed a Hedges’ g of -0.66 (95% CrI: -0.98, -0.35); yoga − 0.68 (-1.21, -0.12); aerobic exercise − 0.56 (-0.79, -0.32); traditional Chinese mind-body exercises − 0.56 (-0.70, -0.42); and multicomponent interventions − 0.47 (-0.64, -0.30). Finally, the effect estimates were similar when stratified by the primary measurement instrument: Hedges’g = -0.56 (95% CrI: -0.74, -0.37) for the ISI and Hedges’g = 0.57 (95% CrI: -0.72, -0.43) for the PSQI. Further details of the subgroup analysis results are provided in the Supplementary Materials.

#### Dose–response relationship

**Fig. 5:**
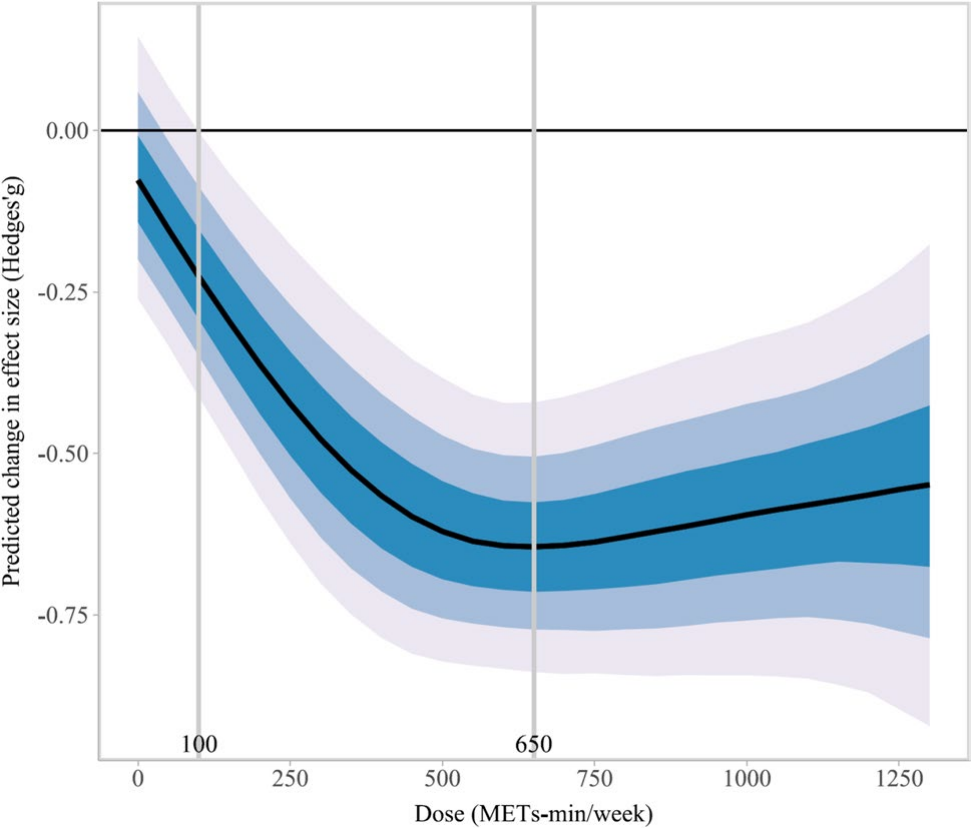
Dose–response curve of physical activity volume and improvement in subjective sleep quality

The dose–response modelling revealed a significant, non-linear “L-shaped” relationship between weekly physical activity volume and improvements in subjective sleep quality, as illustrated in Fig. 5. Specifically, in the lower dosage range, improvements in subjective sleep quality increased steadily with increasing exercise volume. The therapeutic effect peaked at a dose of 650 METs-min/week, with an effect size of Hedges’g = -0.65 (95% CrI: -0.86, -0.45). Beyond this peak, the additional benefit of increasing exercise volume began to wane, demonstrating a trend of diminishing marginal returns. Nevertheless, the effect of physical activity remained significantly superior to that of the control group across all observed dosage levels. Based on this dose–response curve model, the optimal exercise dose predicted to yield the maximal improvement in subjective sleep quality is estimated at 650 METs-mins/week. The minimum effective dose for improving subjective sleep quality was estimated at 100 METs-min/week (defined as the cut-off point where the upper limit of the effect size interval meets 0: Hedges’ g = -0.22, 95% CrI: -0.42, -0.01, with a posterior probability of 95% of achieving this effect).

### Publication bias

**Fig. 6:**
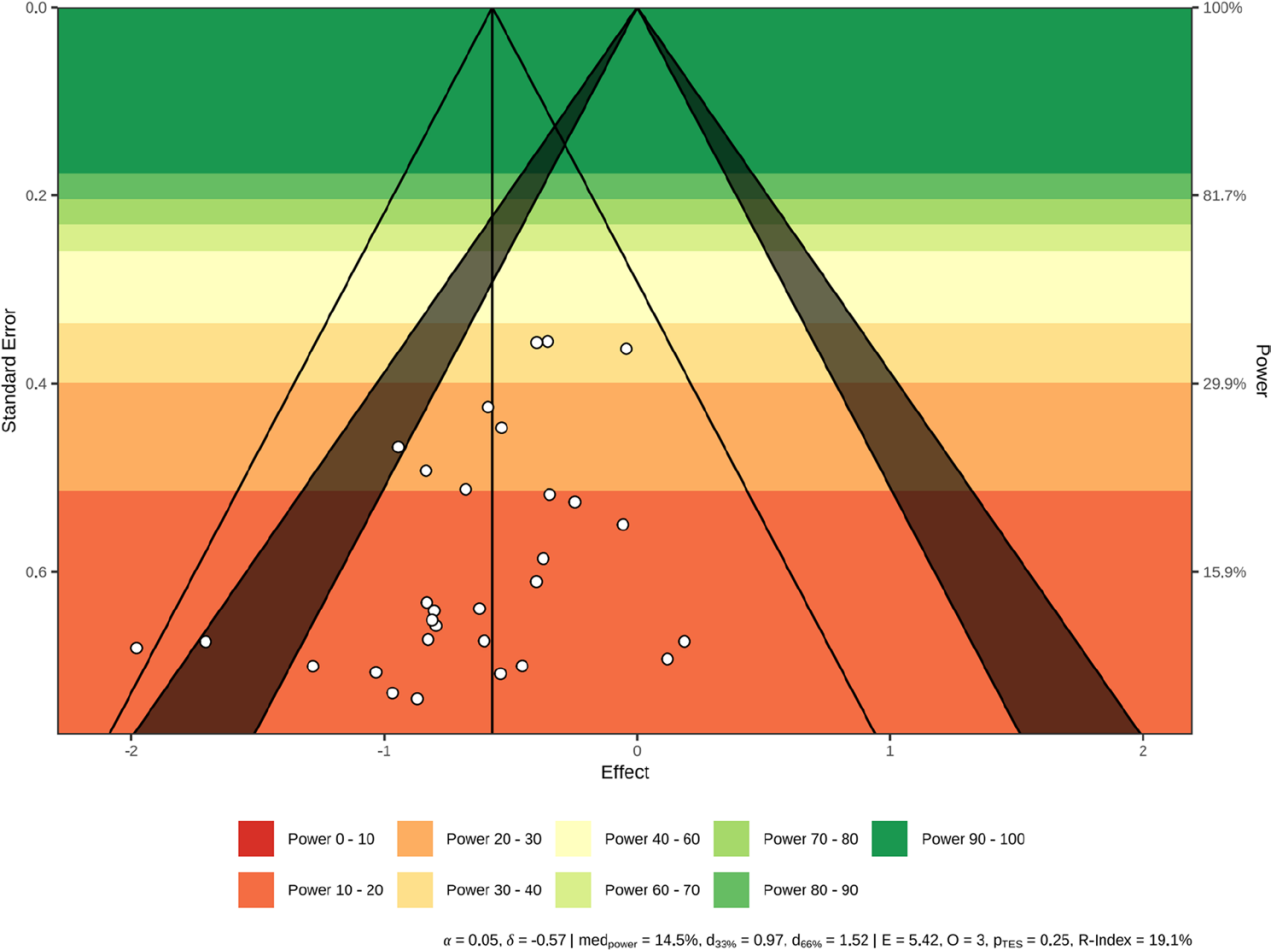
Funnel plot

To assess potential publication bias, a funnel plot was generated (Fig. 6) and Egger’s regression test was performed. Visual inspection of the funnel plot revealed a generally symmetric distribution, with only slight signs of asymmetry characterized by a lack of small studies on the side of smaller effect sizes. However, Egger’s regression test did not reach statistical significance (t = -1.86, *p* = 0.08), suggesting that publication bias is unlikely to have substantially influenced the overall findings. Furthermore, exploratory analysis using the trim-and-fill method did not identify any missing studies requiring imputation.

### Sensitivity analysis

To test the robustness of the findings, pre-specified sensitivity analyses were conducted (Supplemental Materials). Re-fitting the model after excluding the three studies rated as “High risk” of bias revealed no significant changes in the point estimates for either the overall effect of physical activity on subjective sleep quality or the shape of the dose–response curve.

### Certainty of evidence

The overall certainty of evidence for the primary outcome was assessed using the GRADE approach. The results are summarized in Supplemental Materials Table S5. As all evidence in this review was derived from randomized controlled trials, the initial certainty rating was “High”. However, following an assessment of the five domains for potential downgrading, the final certainty of evidence was graded as low for all outcomes.

## Discussion

Through a systematic review of existing RCTs and a Bayesian dose–response meta-analysis, this study quantitatively delineates the relationship between physical activity dosage and improvements in subjective sleep quality among patients with insomnia. It is crucial to note that this meta-analysis deliberately focused on subjective sleep quality as the primary outcome. While objective sleep parameters provide valuable physiological data, insomnia is fundamentally defined by a subjective complaint of unsatisfactory sleep despite adequate opportunity [66]. Therefore, patient self-reported sleep quality represents the most clinically relevant endpoint for evaluating interventions. The findings of this study not only corroborate the overall efficacy of physical activity as a therapeutic intervention but, crucially, reveal a significant, non-linear “L-shaped” dose–response relationship. This curve clearly defines the minimum effective dose for improving subjective sleep quality at approximately 100 METs-min/week and the optimal therapeutic dose at 650 METs-min/week. Beyond this threshold of 650 METs-min/week, increasing exercise volume does not yield significant additional sleep benefits. These findings provide a robust scientific basis for prescribing exercise interventions.

Previous research has established that physical activity effectively improves sleep quality across various age groups [67–70], a conclusion supported by the present study. Our results indicate that physical activity generates a moderate overall improvement in subjective sleep quality for patients with insomnia. When determining the optimal regimen, the choice of exercise modality is a core consideration. Numerous studies, employing direct comparisons or network meta-analyses (NMA), have evaluated the comparative efficacy of aerobic exercise, resistance training, combined protocols, and mind-body exercises (e.g., yoga, Tai Chi, Qigong). A large-scale NMA encompassing 86 RCTs found that all tested modalities—including aerobic, resistance, combined, yoga, Pilates, and traditional Chinese sports—significantly improved sleep quality compared to controls [22]. In terms of Surface Under the Cumulative Ranking Curve rankings, Pilates (91.7%) and aerobic exercise (69.7%) emerged as the most effective [22]. Another NMA confirmed that while mind-body exercises may be superior for subjective sleep quality (measured by PSQI), aerobic exercise is more effective for improving objective sleep efficiency [71]. Consistent with these precedents, our sub-group analysis suggests that while multiple forms of exercise are effective, aerobic exercise (e.g., brisk walking, jogging) yields the most pronounced improvement, followed by resistance training and mind-body exercises.

A critical contribution of this study is the clarification of the specific dose range for patients with insomnia. This finding adds a vital dimension to the existing literature, particularly regarding the prescription of optimal exercise volumes. The “L-shaped” curve identified here likely reflects the intrinsic adaptation patterns of this specific clinical population. Patients with insomnia typically present with baseline subjective sleep quality far below that of the general population, implying substantial room for improvement [22]. Consequently, even relatively low doses of physical activity are sufficient to trigger significant physiological and psychological regulation, resulting in rapid and substantial improvements—constituting the steep ascent of the “L-shaped” curve. However, as subjective sleep quality normalizes, the potential for further improvement naturally diminishes, causing the efficacy curve to plateau. This trajectory contrasts with findings in other populations. A study on non-linear dose–response models in older adults identified an inverted “U-shaped” relationship between physical activity volume and improvements in subjective sleep quality [72]. This implies that as exercise volume increases, the sleep improvement effect initially enhances, reaching a peak; however, if exercise volume continues to increase, the effect may conversely diminish. That study determined that the optimal effect occurs at an exercise volume of approximately 527 METs-min/week [72]. This finding highlights the importance of moderation, even relatively low exercise doses can generate significant sleep benefits, whereas excessive exercise may be ineffective or even counterproductive. Another broader dose–response analysis targeting the general population similarly found a non-linear relationship between exercise dose and sleep quality improvement, presenting an overall “U-shaped” curve [22]. This study indicated that the optimal overall exercise dose is approximately 920 METs-min/week. Similarly, a study by Xue et al. focusing on older adults found a “J-shaped” non-linear relationship between exercise dose and sleep quality improvement, with an optimal overall dose of approximately 440 METs-min/week and a minimum PA dose of about 195 METs-min/week [23]. In the present study, an approximate “L-shaped” dose–response curve was identified in the clinical insomnia population, and the specific dosages also showed distinct differences. These discrepancies collectively point to a deeper conclusion: the dose effect of physical activity on sleep is not universal but is moderated by baseline population characteristics. For the general population, whose sleep typically falls within normal ranges, physical activity serves an optimizing function. While moderate exercise enhances sleep, excessive volume may introduce sleep-disrupting stressors—such as physiological overload, sustained sympathetic hyperarousal triggered by high-intensity exercise near bedtime, or overtraining risks [73–75]—thereby precipitating a “U-shaped” decline in efficacy. For older adults, limited physiological reserves and reduced tolerance for high-intensity exertion imply that peak benefits occur within a lower dosage range; exceeding this threshold may induce fatigue or musculoskeletal discomfort that negates potential gains. In contrast, the core pathology in patients with insomnia is characterized by significant sleep disturbance and a state of hyperarousal [76, 77]. Physical activity functions here as a potent corrective intervention. Even low-to-moderate doses are sufficient to enhance homeostatic sleep drive and attenuate hyperarousal [78]. Once sleep regulatory mechanisms are restored to a relatively normal state, the marginal utility of additional exercise diminishes, resulting in the observed “L-shaped” plateau.

The efficacy of physical activity in treating insomnia stems from its ability to systematically regulate core pathophysiological mechanisms through multiple, interconnected biological and psychological pathways. Physiologically, physical activity profoundly influences the circadian system and thermoregulation. Regular exercise enhances the amplitude of circadian rhythms, promoting daytime alertness and facilitating sleep onset at night, thereby improving sleep continuity and depth [79]. Furthermore, exercise modulates the neuroendocrine system and neurotransmitter activity. It regulates hormone axes related to stress and arousal, particularly the hypothalamic-pituitary-adrenal axis. Dysregulation of cortisol rhythms is central to the sleep-wake cycle [80]; regular aerobic exercise has been shown to lower resting cortisol and improve HPA feedback sensitivity, mitigating the impact of chronic stress [81, 82]. Exercise may also enhance the nocturnal secretion rhythm of melatonin, the sleep-promoting hormone [83–87]. At the neurotransmitter level, exercise increases brain levels of serotonin and gamma-aminobutyric acid, inhibitory neurotransmitters that promote sedation [82, 88], while also modulating the dopamine system involved in arousal and reward [88]. Additionally, exercise-induced release of Brain-Derived Neurotrophic Factor supports neuroplasticity and may indirectly bolster the robustness of sleep regulatory centers [89]. Psychologically, emotion regulation is paramount. Physical activity is a proven anxiolytic and antidepressant, alleviating the negative affective states that precipitate and perpetuate insomnia [90, 91]. By reducing perceived stress and enhancing subjective well-being, exercise creates a psychological environment conducive to restful sleep [92, 93].

This study possesses several strengths. First, the exclusive inclusion of RCTs ensures the validity and reliability of the findings. Second, the application of Bayesian dose–response meta-analysis allows for probabilistic interpretations of the data, offering posterior distributions for all parameters, which enhances the interpretability of the results compared to traditional frequentist methods. However, methodological limitations remain. The primary outcome relied on self-reported measures; while clinically valuable, these are susceptible to placebo effects, expectancy effects, and recall bias. Additionally, despite the explanatory power of the dose–response model, residual heterogeneity exists, likely stemming from variations in intervention protocols, participant characteristics, and control conditions. Finally, this study could not evaluate the long-term sustainability of exercise effects or adherence; future research should address these aspects to fully understand the longitudinal benefits of exercise interventions.

## Conclusions

This Bayesian dose–response meta-analysis provides compelling evidence that physical activity significantly improves subjective sleep quality in patients with insomnia. By precisely mapping the dose–response curve, we identified a minimum effective dose of 100 METs-min/week and an optimal therapeutic dose of 650 METs-min/week. These findings offer a scientific framework for the development of evidence-based, personalized exercise prescriptions for insomnia management. While the results should be interpreted with appropriate caution given the inherent limitations, they strongly support the integration of structured physical activity into standard care. Future large-scale RCTs are warranted to further elucidate the nuances of exercise dosing and its long-term impact on sleep health.

## Supplementary Information

Supplementary Material 1.

## Data Availability

The raw data supporting the conclusions of this article are available from the corresponding author on reasonable request.

## Abbreviations

AHDH: Attention Deficit Hyperactivity Disorder
ASD: Autism Spectrum Disorder
BDNF: Brain-Derived Neurotrophic Factor
CBT-I: Cognitive Behavioral Therapy for Insomnia
CrI: Credible Interval
DSM: Diagnostic and Statistical Manual of Mental Disorders
GRADE: Grading of Recommendations Assessment, Development and Evaluation
HPA: Hypothalamic-Pituitary-Adrenal
HRmax: Maximum Heart Rate
ICSD-3: International Classification of Sleep Disorders, Third Edition
ISI: Insomnia Severity Index
LOOIC: Leave-One-Out Information Criterion
MCMC: Markov Chain Monte Carlo
MeSH: Medical Subject Headings
METs: Metabolic Equivalents
NMA: Network Meta-Analysis
PA: Physical Activity
PICOS: Population, Intervention, Comparator, Outcome, and Study Design
PRISMA: Preferred Reporting Items for Systematic Reviews and Meta-Analyses
PROSPERO: International Prospective Register of Systematic Reviews
PSQI: Pittsburgh Sleep Quality Index
RCT: Randomised Controlled Trial
RoB 2: Cochrane Risk of Bias tool version 2
SD: Standard Deviation
SE: Sleep Efficiency
SMD: Standardised Mean Difference
TCS: Traditional Chinese Sports
VO2max: Maximum Oxygen Uptake
