# Supplementary material for: Dose–response relationship between physical activity and subjective sleep quality in patients with insomnia: a systematic review and Bayesian meta-analysis of randomized controlled trials
Source: Int J Behav Nutr Phys Act. 2026 Apr 11;23:50. doi: 10.1186/s12966-026-01905-0 (PMC13188244; doi:10.1186/s12966-026-01905-0)
Supplement: Supplementary file 1 — Supplementary Material 1. [file 12966_2026_1905_MOESM1_ESM.docx]

**Dose–Response Relationship Between Physical Activity and Subjective Sleep Quality in Patients with Insomnia: A Systematic Review and Bayesian Meta-Analysis**

**Supplemental Materials**

**Table S1 Search strategy**

| #Step | Search Query | Database | Results |
| --- | --- | --- | --- |
| 1 | TS=("Sleep Initiation and Maintenance Disorders" OR insomnia* OR "sleep disorder*" OR "sleep disturbance*" OR "sleep complaint*" OR "poor sleep") | Web of Science | 83942 |
| 2 | TI=("Exercise" OR "Physical Activity" OR exercise* OR "physical activit*" OR sport* OR training OR walking OR jogging OR running OR cycling OR swimming OR yoga OR "tai chi" OR resistance) | Web of Science | 964144 |
| 3 | TS=("Randomized Controlled Trial" OR "Controlled Clinical Trial" OR randomized OR placebo OR randomly OR trial) | Web of Science | 2120369 |
| 4 | #1 AND #2 AND #3 | Web of Science | 872 |
| 4 | #1 AND #2 AND #3 | PubMed | 2,872 |
| 3 | "Randomized Controlled Trial"[pt] OR "Controlled Clinical Trial"[pt] OR randomized[tiab] OR placebo[tiab] OR randomly[tiab] OR trial[tiab] OR groups[tiab] | PubMed | 4,379,908 |
| 2 | "Exercise"[Mesh] OR "Physical Activity"[tiab] OR exercise*[tiab] OR "physical activit*"[tiab] OR sport*[tiab] OR training[tiab] OR walking[tiab] OR jogging[tiab] OR running[tiab] OR cycling[tiab] OR swimming[tiab] OR yoga[tiab] OR "tai chi"[tiab] OR resistance[tiab] | PubMed | 2,579,710 |
| 1 | "Sleep Initiation and Maintenance Disorders"[Mesh] OR insomnia*[tiab] OR "sleep disorder*"[tiab] OR "sleep disturbance*"[tiab] OR "sleep complaint*"[tiab] OR "poor sleep"[tiab] | PubMed | 97,253 |
| 3 | ('insomnia':ti OR 'insomnia*':ti OR 'sleep disorder*':ti OR 'sleep disturbance*':ti OR 'sleep complaint*':ti OR 'poor sleep':ti) AND ('exercise':ab,ti OR 'physical activity':ab,ti OR 'exercise*':ab,ti OR 'physical activit*':ab,ti OR 'sport*':ab,ti OR 'training':ab,ti OR 'walking':ab,ti OR 'jogging':ab,ti OR 'running':ab,ti OR 'cycling':ab,ti OR 'swimming':ab,ti OR 'yoga':ab,ti OR 'tai chi':ab,ti OR 'resistance':ab,ti) AND ('randomized controlled trial':ti,ab,kw OR 'controlled clinical trial':ti,ab,kw OR 'randomized':ti,ab,kw OR 'placebo':ti,ab,kw OR 'randomly':ti,ab,kw OR 'trial':ti,ab,kw OR 'groups':ti,ab,kw) | Embase | 1212 |
| 2 | 'exercise'/exp OR 'physical activity':ti,ab,kw OR 'exercise*':ti,ab,kw OR 'physical activit*':ti,ab,kw OR 'sport*':ti,ab,kw OR 'training':ti,ab,kw OR 'walking':ti,ab,kw OR 'jogging':ti,ab,kw OR 'running':ti,ab,kw OR 'cycling':ti,ab,kw OR 'swimming':ti,ab,kw OR 'yoga':ti,ab,kw OR 'tai chi':ti,ab,kw OR 'resistance':ti,ab,kw | Embase | 3462076 |
| 1 | 'insomnia'/exp OR 'insomnia' OR 'insomnia*':ti,ab,kw OR 'sleep disorder*':ti,ab,kw OR 'sleep disturbance*':ti,ab,kw OR 'sleep complaint*':ti,ab,kw OR 'poor sleep':ti,ab,kw | Embase | 208519 |
| 1 | ('insomnia' OR 'insomnia*' OR 'sleep disorder*' OR 'sleep disturbance*' OR 'sleep complaint*' OR 'poor sleep'):ab | Cochrane Library | 29723 |
| 2 | ('exercise' OR 'physical activity' OR 'exercise*' OR 'physical activit*' OR 'sport*' OR 'training' OR 'walking' OR 'jogging' OR 'running' OR 'cycling' OR 'swimming' OR 'yoga' OR 'tai chi' OR 'resistance'):ab | Cochrane Library | 320057 |
| 3 | (Randomized Controlled Trial OR Randomized OR Random OR Placebo OR RCT OR Trial OR Clinical Trial):pt | Cochrane Library | 607670 |
| 4 | #1 AND #2 AND #3 | Cochrane Library | 3272 |
| 1 | summary('exercise' OR 'physical activity' OR 'exercise*' OR 'physical activit*' OR 'sport*' OR 'training' OR 'walking' OR 'jogging' OR 'running' OR 'cycling' OR 'swimming' OR 'yoga' OR 'tai chi' OR 'resistance') AND summary('insomnia' OR 'insomnia*' OR 'sleep disorder*' OR 'sleep disturbance*' OR 'sleep complaint*' OR 'poor sleep') AND summary('randomized controlled trial' OR 'controlled clinical trial' OR 'randomized' OR 'placebo' OR 'randomly' OR 'trial') | PsycINFO | 953 |

**Table S2 Subgroup analysis**

| **Subgroup variable** | ***k*** | **Hedges’g 95%CrI** | ***I^2^_within_*** | ***I^2^_between_*** | ***R^2^*** |
| --- | --- | --- | --- | --- | --- |
| **Age** |  |  | 7.24% | 32.92% | 0.60 |
| Adults | 8 | -1.00 [-1.36, -0.64] |  |  |  |
| Older Adults | 15 | -0.51 [-0.64, -0.38] |  |  |  |
| **Diagnostic criteria** |  |  | 7.95% | 35.78% | 0.58 |
| Formal diagnostic criteria | 10 | -0.52 [-0.66, -0.38] |  |  |  |
| Questionnaire cut-off | 14 | -0.66 [-0.87, -0.46] |  |  |  |
| **Baseline insomnia severity** |  |  | 9.97% | 35.35% | 0.59 |
| Mild | 10 | -0.46 [-0.63, -0.29] |  |  |  |
| Moderate | 17 | -0.65 [-0.81, -0.50] |  |  |  |
| **Physical activity modalities** |  |  | 8.35% | 42.21% | 0.61 |
| Yoga | 2 | -0.68 [-1.21, -0.12] |  |  |  |
| Strength | 4 | -0.66 [-0.98, -0.35] |  |  |  |
| Aerobic exercise | 12 | -0.56 [-0.79, -0.32] |  |  |  |
| Traditional Chinese mind-body exercises | 6 | -0.56 [-0.70, -0.42] |  |  |  |
| Multicomponent | 6 | 0.47 [-0.64, -0.30] |  |  |  |
| **Measurement instrument** |  |  | 9.04% | 39.75% | 0.57 |
| PSQI | 19 | -0.57 [-0.72, -0.43] |  |  |  |
| ISI | 12 | -0.56 [-0.74, -0.37] |  |  |  |

**Table S3 Predicted effects on sleep quality by dose**

| Dose | Hedges’g | Lower 95%CrI | Upper 95%CrI |
| --- | --- | --- | --- |
| 0 | -0.085 | -0.285 | 0.114 |
| 50 | -0.162 | -0.36 | 0.035 |
| 100 | -0.228 | -0.42 | -0.019 |
| 150 | -0.311 | -0.521 | -0.1 |
| 200 | -0.379 | -0.6 | -0.158 |
| 250 | -0.442 | -0.672 | -0.212 |
| 300 | -0.498 | -0.734 | -0.262 |
| 350 | -0.545 | -0.783 | -0.308 |
| 400 | -0.584 | -0.818 | -0.35 |
| 450 | -0.614 | -0.841 | -0.387 |
| 500 | -0.635 | -0.853 | -0.417 |
| 550 | -0.648 | -0.857 | -0.439 |
| 600 | -0.652 | -0.858 | -0.449 |
| 650 | -0.653 | -0.858 | -0.449 |
| 700 | -0.648 | -0.858 | -0.438 |
| 750 | -0.64 | -0.859 | -0.421 |
| 800 | -0.63 | -0.859 | -0.402 |
| 850 | -0.62 | -0.858 | -0.383 |
| 900 | -0.61 | -0.855 | -0.366 |
| 950 | -0.601 | -0.851 | -0.351 |
| 1000 | -0.593 | -0.848 | -0.337 |
| 1050 | -0.584 | -0.847 | -0.322 |
| 1100 | -0.577 | -0.85 | -0.304 |
| 1150 | -0.57 | -0.858 | -0.281 |
| 1200 | -0.563 | -0.872 | -0.254 |
| 1250 | -0.556 | -0.891 | -0.222 |
| 1300 | -0.55 | -0.915 | -0.185 |

**Table S4 Sensitivity analysis for overall effect**

| Model | Number of studies | Hedges’g | 95%CrI |
| --- | --- | --- | --- |
| Full model | 24 | -0.56 | [-0.68, -0.45] |
| Sensitivity model | 21 | -0.56 | [-0.68, -0.44] |


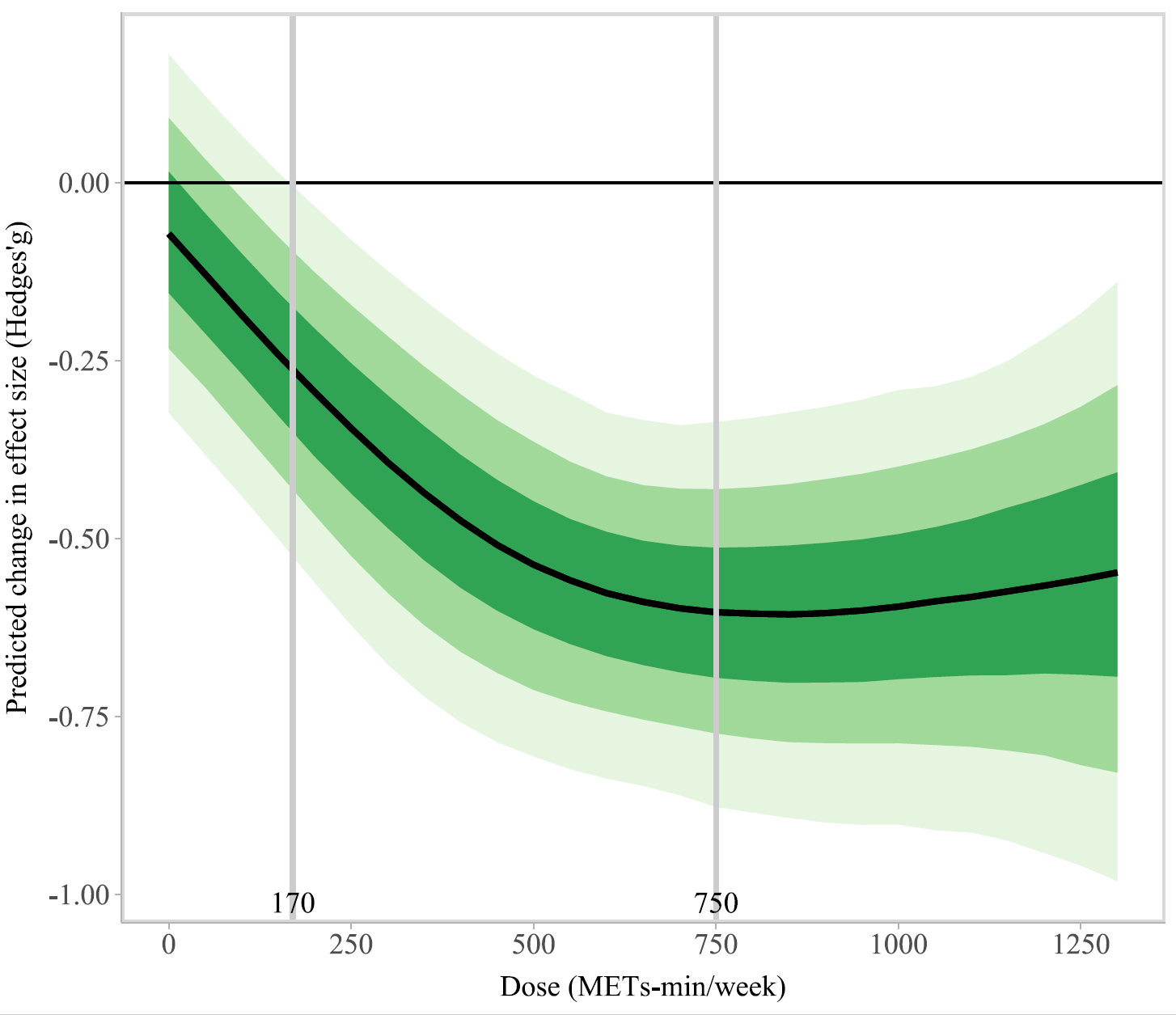


**Figure S1 Sensitivity analysis for dose-response curve**

**Table S5: GRADE Summary of Findings**

| **Outcome** | **Number of studies** | **Study design** | **GRADE Assessment** | | | | | **Effect (SMD [95% CrI])** | **Certainty of Evidence** |
| --- | --- | --- | --- | --- | --- | --- | --- | --- | --- |
|  |  |  | **Risk of bias** | **Inconsistency** | **Indirectness** | **Imprecision** | **Publication bias** |  |  |
| **sleep quality** | 24 | Randomized trials | Serious | Not serious | Not serious | Serious | Not serious | -0.56 [-0.68 to -0.45] | ⨁⨁◯◯ Low |
| Risk of bias: Among the included studies, six (out of twenty) were rated as having "some concerns" or "high risk" of bias. This stemmed primarily from the unavoidable lack of blinding in behavioral interventions and potential performance or detection bias associated with subjective outcome measures. These systematic risks posed a serious threat to the validity of the effect estimate. Therefore, the certainty was downgraded by one level.  Inconsistency: Not downgraded. Although statistical heterogeneity across studies was high, this heterogeneity could be largely explained by variations in exercise dose between studies, which is precisely the issue the dose-response analysis aimed to address. Sensitivity analyses also indicated that the shape of the dose-response relationship was robust.  Indirectness: Not downgraded. The populations, interventions, comparators, and outcomes in the included studies directly corresponded to the PICOS question of this review.  Imprecision: Downgraded by one level. The dose-response curve and its 95% credible interval, as well as the estimate of the optimal dose, showed a concerning lack of precision. Beyond approximately 1200 METs, the shaded 95% CI region became very wide and even crossed the line of no effect. This indicates that the evidence is imprecise, particularly in the higher dose range.  Publication bias: Not downgraded. Formal statistical testing did not provide strong evidence for the presence of substantial publication bias.  *CI, credible interval; SMD, standardized mean difference | | | | | | | | | |
